# Supplementary material for: Maternal and Infant Histo-Blood Group Antigen (HBGA) Profiles and Their Influence on Oral Rotavirus Vaccine (RotarixTM) Immunogenicity among Infants in Zambia
Source: Vaccines (Basel). 2023 Jul 31;11(8):1303. doi: 10.3390/vaccines11081303 (PMC10458424; doi:10.3390/vaccines11081303)
Supplement: Supplementary file 1 [file vaccines-11-01303-s001.zip › S1_Supplementary information_Data_12072023.pdf]

Supplementary information. Material and Methods

1. Supplementary Data

Genotypic characterization (G428A) of FUT2 genes. Genomic DNA was extracted from samples (200 µL saliva) using a QIAgen DNA mini kit (Qiagen), following manufacturer's instructions. A 1125 bp long fragment containing the entire coding region (1032 bp, exon 2) of the FUT2 gene was amplified by PCR from template genomic DNA using previously published primers, FUT2-F and FUT2-R (Ferrer-Admetlla, 2009). Using extracted genomic DNA, PCR was used to amplify a 1125 base pair (bp) fragment containing the entire coding region (1032 bp, exon 2) of the FUT2 gene based on published primers (Ferrer-Admetlla, 2009) listed in Table 1.

Table 1. Primer list for FUT2 genotyping

Primer Name	Primer Sequence
FUT2-FOR1	CCATCTCCCAGCTAACGTGTCC
FUT2-REV1	GGGAGGCAGAGAAGGAGAAAAGG

Note: FR1 = First Forward Primer; RV1 = First Reverse Primer; FOR1=Forward primer; REV1= Reverse Primer

A 25 µL PCR reaction containing 10X PCR buffer, 10 mM deoxynucleotide triphosphate (dNTP) mix, Platinum Taq DNA polymerase, 20 µM forward and reverse primer and genomic DNA extract was run on a Gene Amp® PCR System 9700 thermocycler under the following conditions: 96°C, 10 mins; 94°C, 30 secs, 59°C, 30 secs, 72°C, 90 secs [35 cycles]; 72°C, 10 mins. PCR amplicons were confirmed by electrophoresis of PCR products on 1.5% agarose gel stained with gel red alongside a 100 bp molecular marker and visualization of bands was performed under ultra-violet (UV) light.

FUT2 G428A genotypes were determined based on RFLP patterns:

<u>**Key for interpretation of results**</u>

FUT2 G428A genotypes are determined based on RFLP patterns:

- **Digestion into 3 fragments: 202 bp, 425 bp, and 498 bp was recorded as Genotype AA; *Non-secretor***
- **Digestion into 4 fragments: 202 bp, 295 bp, 130 bp and 498 bp was recorded as Genotype GG; *Homozygous secretor***
- **Digestion into 5 fragments: 202 bp, 295 bp, 130 bp, 425 bp and 498 bp was recorded as Genotype GA; *Heterozygous secretor***

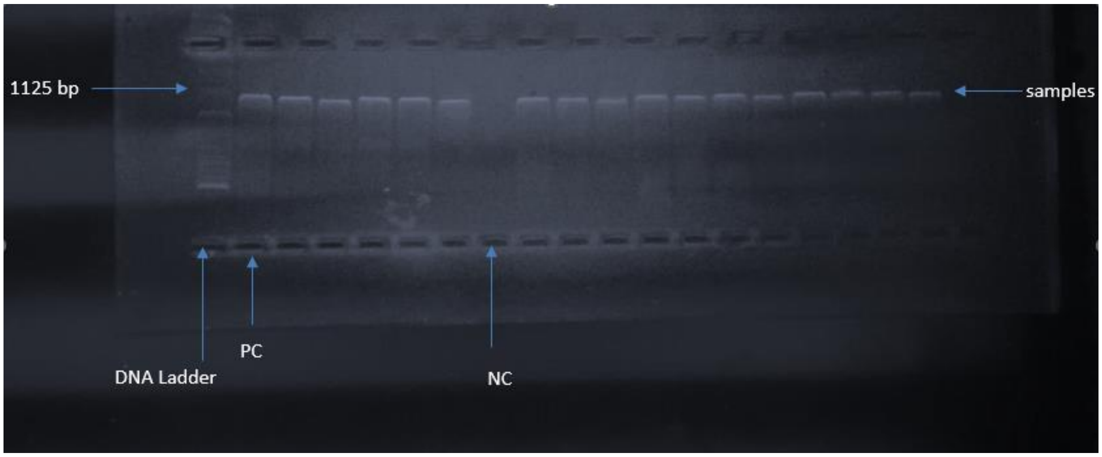

Figure 1. FUT2 Amplicons on Agarose gel

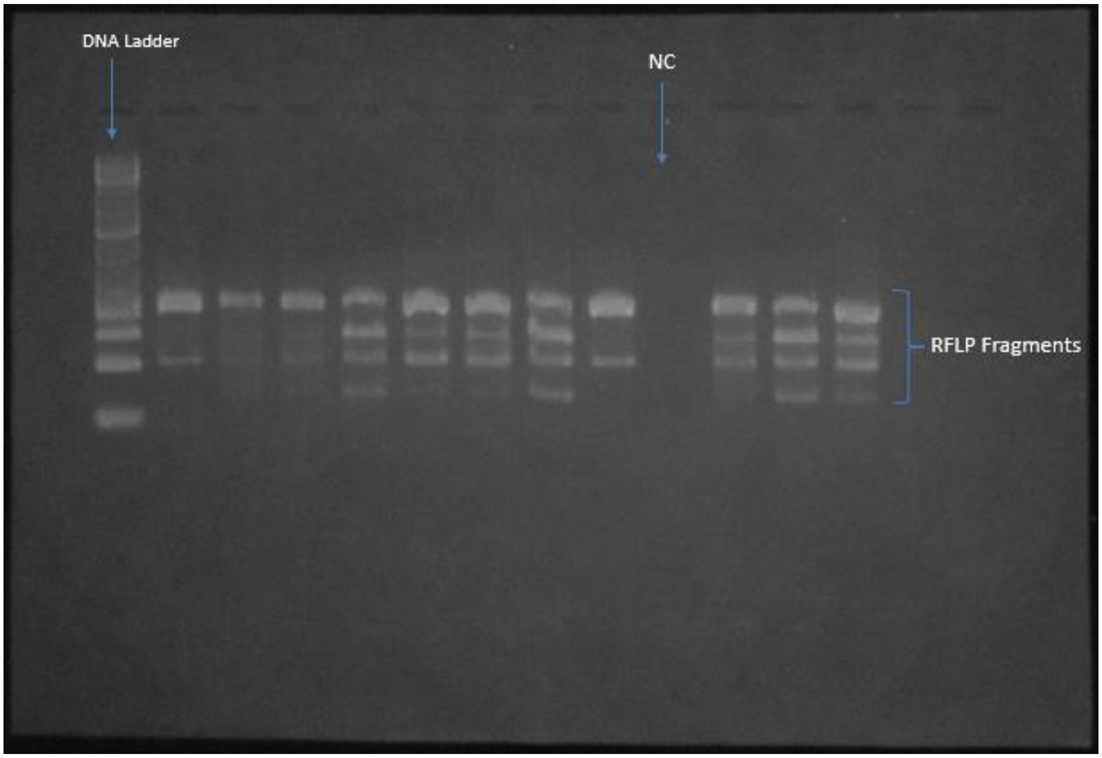

Figure 2. FUT2 RFLP Fragments on agarose gel
