# Supplementary material for: Maternal and Infant Histo-Blood Group Antigen (HBGA) Profiles and Their Influence on Oral Rotavirus Vaccine (RotarixTM) Immunogenicity among Infants in Zambia
Source: Vaccines (Basel). 2023 Jul 31;11(8):1303. doi: 10.3390/vaccines11081303 (PMC10458424; doi:10.3390/vaccines11081303)
Supplement: Supplementary file 1 [file vaccines-11-01303-s001.zip › S2_Supplementary information_Tables and figures_12072023.pdf]

Supplementary information. Material and Methods

Table 1. Primer list for FUT2 genotyping

Primer Name	Primer Sequence
FUT2-FOR1	CCATCTCCCAGCTAACGTGTCC
FUT2-REV1	GGGAGGCAGAGAAGGAGAAAAGG

Note: FR1 = First Forward Primer; RV1 = First Reverse Primer; FOR1 = Forward primer; REV1 = Reverse Primer

Key for interpretation of results

FUT2 G428A genotypes are determined based on RFLP patterns:

- **Digestion into 3 fragments: 202 bp, 425 bp, and 498 bp was recorded as Genotype AA; *Non-secretor***
- **Digestion into 4 fragments: 202 bp, 295 bp, 130 bp and 498 bp was recorded as Genotype GG; *Homozygous secretor***
- **Digestion into 5 fragments: 202 bp, 295 bp, 130 bp, 425 bp and 498 bp was recorded as Genotype GA; *Heterozygous secretor***

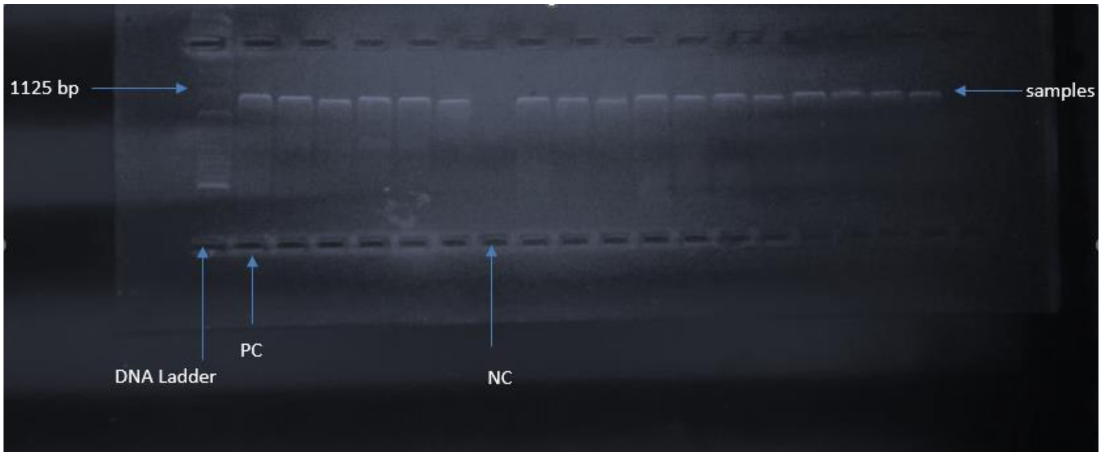

Figure 1. FUT2 Amplicons on Agarose gel

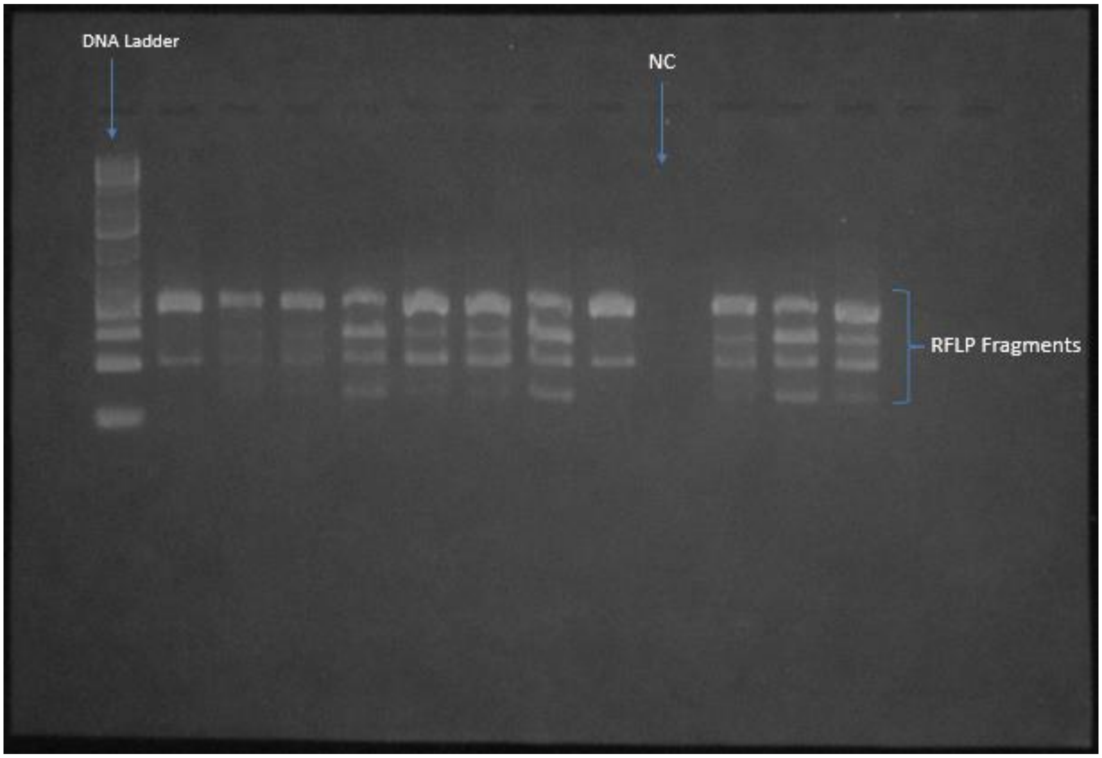

Figure 2. FUT2 RFLP Fragments on agarose gel

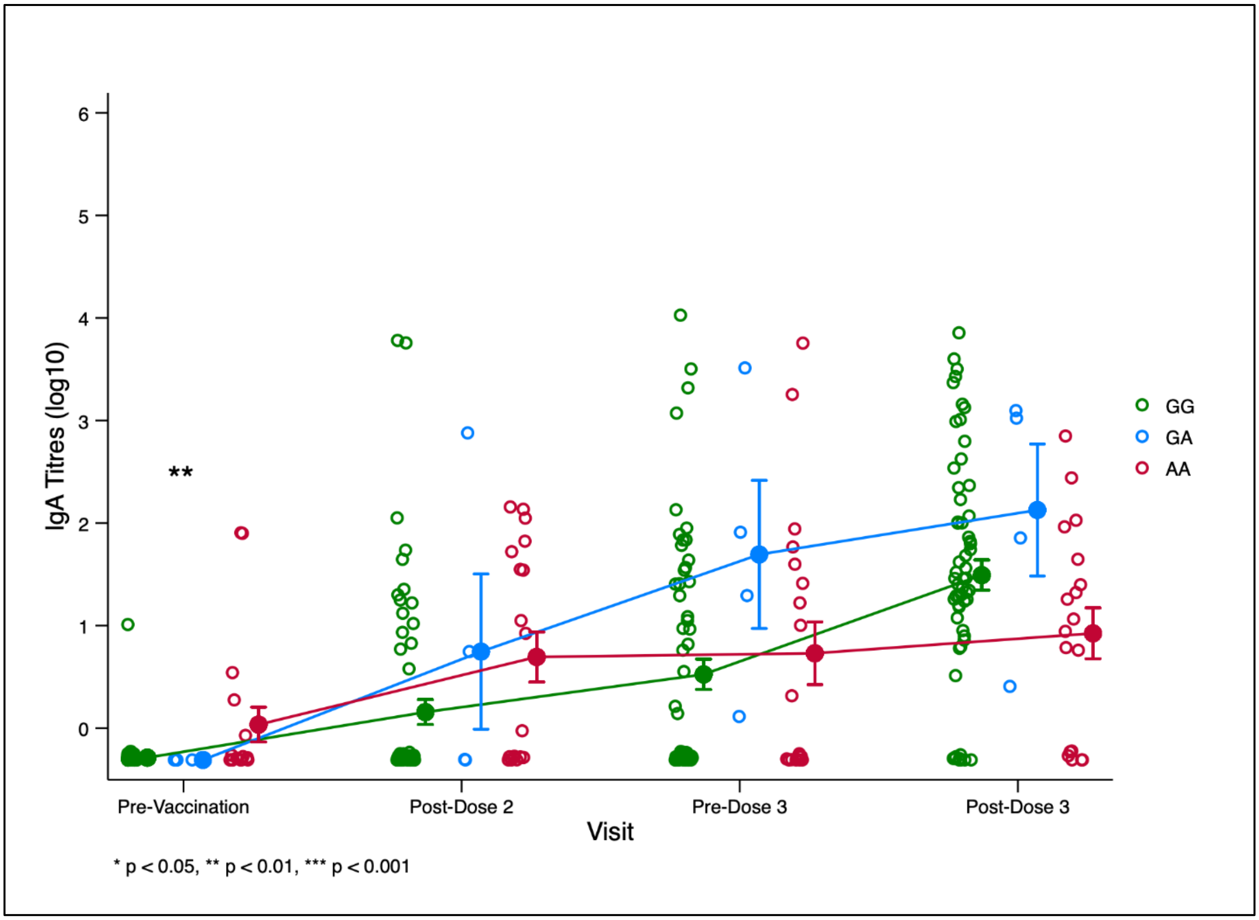

Figure 3c.Trend plot for infant RV-IgA titre kinetics for Lewis phenotype. The green and marron lines represent the trends of RV-IgA titres over time for the Lewis-null (Le-) and Lewis-positive (Le+) phenotypes respectively.*** p<0.001
